# The Non-Equilibrium Thermodynamics and Kinetics of Focal Adhesion Dynamics

**DOI:** 10.1371/journal.pone.0012043

**Published:** 2010-08-18

**Authors:** Joseph E. Olberding, Michael D. Thouless, Ellen M. Arruda, Krishna Garikipati

**Affiliations:** 1 Department of Biomedical Engineering, University of Michigan, Ann Arbor, Michigan, United States of America; 2 Departments of Mechanical Engineering, and Materials Science and Engineering, University of Michigan, Ann Arbor, Michigan, United States of America; 3 Department of Mechanical Engineering and Program in Macromolecular Science and Engineering, University of Michigan, Ann Arbor, Michigan, United States of America; 4 Department of Mechanical Engineering, Michigan Center for Theoretical Physics and Center for Computational Medicine and Bioinformatics, University of Michigan, Ann Arbor, Michigan, United States of America; Massachusetts Institute of Technology, United States of America

## Abstract

**Background:**

We consider a focal adhesion to be made up of molecular complexes, each consisting of a ligand, an integrin molecule, and associated plaque proteins. Free energy changes drive the binding and unbinding of these complexes and thereby controls the focal adhesion's dynamic modes of growth, treadmilling and resorption.

**Principal Findings:**

We have identified a competition among four thermodynamic driving forces for focal adhesion dynamics: (i) the work done during the addition of a single molecular complex of a certain size, (ii) the chemical free energy change associated with the addition of a molecular complex, (iii) the elastic free energy change associated with deformation of focal adhesions and the cell membrane, and (iv) the work done on a molecular conformational change. We have developed a theoretical treatment of focal adhesion dynamics as a nonlinear rate process governed by a classical kinetic model. We also express the rates as being driven by out-of-equilibrium thermodynamic driving forces, and modulated by kinetics. The mechanisms governed by the above four effects allow focal adhesions to exhibit a rich variety of behavior without the need to introduce special constitutive assumptions for their response. For the reaction-limited case growth, treadmilling and resorption are all predicted by a very simple chemo-mechanical model. Treadmilling requires symmetry breaking between the ends of the focal adhesion, and is achieved by driving force (i) above. In contrast, depending on its numerical value (ii) causes symmetric growth, resorption or is neutral, (iii) causes symmetric resorption, and (iv) causes symmetric growth. These findings hold for a range of conditions: temporally-constant force or stress, and for spatially-uniform and non-uniform stress distribution over the FA. The symmetric growth mode dominates for temporally-constant stress, with a reduced treadmilling regime.

**Significance:**

In addition to explaining focal adhesion dynamics, this treatment can be coupled with models of cytoskeleton dynamics and contribute to the understanding of cell motility.

## Introduction

A focal adhesion (FA) is a type of cell-substrate attachment mediated by bonds between the transmembrane protein, integrin, and an extracellular matrix (ECM) protein such as fibronectin. In this communication, we consider FAs in fibroblasts, although they are observed in other mesenchymal cell types as well. The integrins in FAs are believed to associate with over 50 cytoplasmic “plaque proteins” [1] among which are tensin, paxillin [2], [3], vinculin [2]–[4], talin and zyxin [3]. Many of these proteins have been detected in fluorescence studies of FAs. Intracellular attachments are formed to actin stress fibers [4] by the association of vinculin and talin to the FAs, followed by the binding of these proteins to F-actin [5]–[7]. It has been demonstrated by numerous experiments that force can be transmitted to FAs by the actin stress fibers. The force can be generated either by actomyosin contractility, or by an external manipulation such as with a micropipette [2], [4], [8]. The FAs transmit this force to the ECM. FA growth and resorption is strongly dependent on this force, as shown by a number of studies [2], [4], [9], [10].

In fibroblasts, a rich dynamic behavior of FAs is obtained when they are subject to force [11]. The FAs grow into elongated structures at a rate of 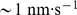 [2]. They tend to be elongated in the cell-substrate interfacial plane with the long axis aligned with the force component in this plane. In the case of cell-generated tension the FA area is proportional to the force on it with a stress 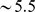 kPa [4].

FA dynamics have been visualized via fluorescence in cells that are contracting or loaded by external force [8], [12]. These studies show unbinding of proteins at the ends that are distally-located with respect to the stress fiber or point of application of external force. The associated “peeling” has been studied by mounting cells and synthetically-constituted vesicles on substrates, and modelled by applying methods of fracture mechanics extended to reaction-diffusion systems [13], [14]. However, the aspect of FA dynamics that has attracted the attention of experimental cell biologists and theoretical biophysicists alike is the force-mediated growth of the proximal end by the binding of proteins—an observation that is surprising because it runs counter to intuition gained from fracture processes such as tape peeling. The relative velocities of the proximal and distal ends combine to create different regimes of FA dynamics: growth at both ends, treadmilling that consists of growth at one end due to binding and resorption at the other end due to unbinding [2], [8], and resorption at both ends. The directions of growth, treadmilling and resorption are aligned with the force [2], [8], [12]. Treadmilling is directed toward the attached stress fiber when induced by actomyosin contractility, and is directed along the external force otherwise. Under such force-mediated growth, “focal complexes”, typically observed at sizes 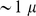
m [12], mature into larger structures recognized as FAs.

Fluorescence studies reveal the binding and unbinding of labelled proteins, but do not explain the biophysics of FA growth under force. Some experimental papers have aimed to explain this growth by hypothesizing molecular mechanisms such as the exposure of cryptic self-association sites on fibronectin by the applied tensile force that is transmitted to fibronectin after formation of integrin-fibronectin bonds [6]. This hypothesis suggests that tension causes a conformational change in the fibronectin molecule, exposing a previously cryptic site, and enabling polymerization of the ECM's fibronectin network. In turn, this allows more integrins to bind to the fibronectin network, and promotes FA growth. A tension-induced conformational change also has been hypothesized for vinculin activation during assembly of FA plaque proteins [1].

A few theoretical studies [8], [15]–[19] have sought to explain the biophysics of FA growth based on the above experimental observations. Of interest for the general mathematical treatment of adhesion of cellular structures driven by strain energy and chemistry is the work of Freund and Lin [20]. More recent studies have considered the strength of receptor-ligand binding in a statistical mechanics setting [21], the clustering of receptor-ligand bonds via a stability analysis [22], and the evolution of a bond over a free energy landscape under influence of a force [23]. A recent molecular dynamics study [7] has attempted to shed light on the force-induced conformational change of talin that enables its binding with vinculin.

In this work we treat FA dynamics as a rate process governed by Harmonic Transition State Theory [24]. Starting from this basis, we write the rates as being driven by out-of-equilibrium thermodynamic driving forces, and modulated by kinetics. We also make a connection to classical non-equilibrium thermodynamics with linear response theory as laid out by de Groot and Mazur [25]. We consider a number of mechanisms, both chemical and mechanical, that affect the out-of-equilibrium thermodynamic driving forces. We frame the discussion in terms of the symmetry, with respect to distal and proximal ends, that each mechanism imposes on the thermodynamic driving forces. Our central finding is that one contribution to the work done by actin stress fiber-transmitted force is antisymmetric and enables the treadmilling mode of FA dynamics, and that once the symmetry is broken, it can be further skewed by a force-enhanced kinetic effect. Other chemical and mechanical effects cause symmetric growth or resorption.

## Analysis

**Figure 1:**
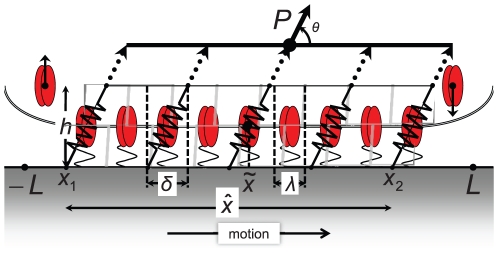
Physical and mathematical model of a FA. The FA is the grey-colored parallelogram. Binders are shown as double ellipses, either free or bound in complexes. The bound complexes have been depicted to be larger, of length 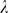 in the 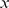-direction and their elastic response is represented by the springs. The elastic elements have length 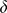 along the 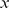-direction. (Note that 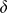 is an arbitrary length and has no effect on the potential; see the section titled “Driving force due to elasticity”.) The dotted arrows are actin stress fibers, which transfer force to the attached complexes. The bundle of actin stress fibers transmits total force 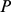. Also shown are the proximal and distal ends, 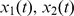, the centroid 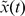, length 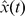 and domain boundaries 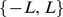.

Fig. 1 represents an FA experiencing proximal end growth via binding, and distal end resorption via unbinding under a tensile force transmitted by the actin stress fibers. The binding and proximal end growth of FAs is intriguing for its force-dependence, instances of which have been cited in the Introduction. This suggests that, while chemistry has a central role by the very fact that binding and unbinding are taking place, mechanical forces are able to influence the dynamics to a significant degree. We seek to explain this coupling of chemistry and mechanics (chemo-mechanics) in terms of non-equilibrium thermodynamics and kinetics.

### Physical aspects

#### Consideration of forces that are constant in time

Absent any experimental interventions, the total force transmitted to an FA by the actin stress fibers can be time-dependent due to its coupling with actomyosin contractility and the dynamics of stress fibers. Balaban and co-workers [4] used patterned elastomeric substrates to target single FAs in stationary human foreskin fibroblasts. They found the force on a FA to be linearly related to its area, with a stress of 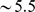 kPa on the dynamically growing/resorbing FAs. However, in earlier work Galbraith and Sheetz [26] estimated that in chicken embryo fibroblasts migrating on a surface patterned with micromachined pads, the force generated by a single FA was 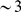 nN. Their observation time frame was of the order of 1800 s, during which time the cells migrated over distances of 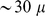
m. This suggests two conclusions: (a) During cell locomotion, the system consisting of actin stress fibers attached to a single FA is capable of maintaining a roughly constant force over 1800 s. (b) The FA dynamics that interests us may have been taking place in these experiments but remained unobserved since the cell migration velocities of 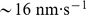 dominate FA growth velocities of 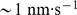 [2]. These conclusions suggest additionally that it would be of interest to study FA dynamics under constant force. Using the methods of Shen and co-workers [27] it appears that it would be feasible to load individual FAs by micropillar actuation and subject them to constant-in-time force control. In such an experiment, the law of mechanical equilibrium would require that the balancing force from stress fibers on the FA remain constant in time. Motivated thus, we consider the dynamics of a single FA under constant total force, 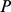, as a means to probe the biophysics. We also note that at constant force and temperature the Gibbs free energy is the relevant thermodynamic potential, and it allows some simplification of the mathematical treatment in this preliminary study. Since we consider the case of constant force 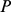, the elasticity of actin stress fibers does not enter our formulation. Therefore we do not model the actin stress fibers explicitly, but we note that their effect is represented by the total force, 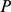, transmitted by the fibers.

Our treatment remains applicable if the stress is constant in time. This case has been worked out in the appendix. The greater complexity entailed by a force that varies in time with the dynamics of actomyosin contractility and stress fiber remodelling will be the subject of a subsequent study. The physical mechanisms that are identified in this work may remain valid in that situation, but their specific actions will probably differ.

#### A reaction-limited process

The formation of a bound complex requires diffusion of integrins in the cell membrane to a ligand binding site, followed by integrin-ligand binding, and subsequent diffusion of plaque proteins to the integrin site for formation of the integrin-ligand-plaque protein complex. Once formed, these complexes appear to be immobile in the interfacial plane of the cell membrane and substrate. The concentration of complexes will be denoted by 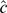. Diffusion and reaction-limited regimes have been considered for cell adhesion by Bell [28], although not explicitly for FA dynamics. Here we restrict ourselves to the reaction-limited regime by considering the diffusion of integrin and plaque proteins to happen relatively fast. The FA is able to exchange mass via binding and unbinding with molecules from the reservoirs of proteins in the cell membrane and cytosol, and the substrate reservoir of free ligands. These reservoirs are considered to remain at equilibrium. For concentration-dependent potentials of cytosolic proteins and free ligands, the implication is that these species can be represented by a single free binder species at fixed concentration, 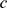. The FA itself is not at equilibrium and is able to dynamically grow and undergo resorption (see File S1 and Movies S1, S2, S3, S4, S5, S6, S7, S8). Our computations confirm this setting of the reaction-limited regime to be a relevant limiting case, and a good model for the more detailed reaction-diffusion problem. We have provided computations of the full reaction-diffusion formulation as supporting information (File S1; Movies S9 and S10).

#### The FA geometry is predominantly one-dimensional

Images and supplementary information from several studies [1], [2], [4], [8], [11] have not indicated any noticeable variation in the FA width even as it grows and shrinks in length due to binding/unbinding of ligands, integrins and plaque proteins. More recent work has shown some width-wise variation, but it is significantly less than changes in length [10]. Motivated thus, we suppose that the FA maintains its width, say 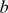, even as its length changes. (However, also see the section titled “Summary of results in relation to mechanisms” where the tendency for weaker widthwise growth is explained in terms of this model.) The geometry and dynamics being predominantly one-dimensional, 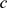 and 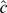 will both be given units of numbers per unit length. Also, as discussed above, 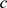 is taken to be an equilibrium value.

#### Bounds on the size of a complex

Arnold and co-workers [29] studied osteoblast attachment on substrates that were deposited with protein-functionalized gold nanodots of 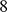 nm diameter for inter-nanodot separations of 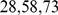 and 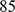 nm. Their studies showed that FAs fail to develop if the separation between nanodots exceeds 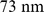. We interpret this result to imply that there is a maximum allowed spacing between bound complexes in the lengthwise direction above which the FA does not form. This maximum value will be denoted by 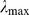 in our model. As the minimum spacing, 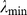, we use the integrin molecule packing separation of 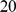 nm [30].

#### The elasticity model

We exploit the fact that FA formation involves integrin-ligand, integrin-plaque protein and plaque protein-actin bonds, and that the dominant direction of bonding is perpendicular to the cell membrane-substrate interface in the absence of force. While some bonds are understood to be formed between plaque proteins, it is not clear that there is sufficient lateral bonding to confer some shear stiffness on the FA. We will neglect the shear stiffness. Our mechanical model of the FA is therefore not an elastic layer, but a row of elastic elements arrayed in the lengthwise direction, each of which can only resist force along the ligand-integrin-plaque protein axis (Fig. 1). Bell [28] made the same observation on the deformation mechanism in adhesion bonds. We consider elastic elements, each of size 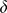. Consider an elastic element located at some point along the FA, and suppose that this element contains 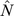 bound complexes. The average size of a complex within this elastic element is 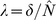, and therefore the concentration averaged over the element is 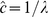. The bounds introduced above for complex size lead to bounds on the concentration: 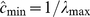, and 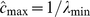. Here, 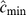 is the concentration below which any assemblage of molecules is not considered to constitute an FA. The elastic element has a concentration-dependent Young's modulus 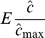. Following the elementary model of a linear elastic rod of cross-sectional area 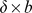 and height 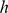, the stiffness of each elastic element along the ligand-integrin-plaque protein axis is 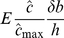. (Also see the treatment of bond elasticity by Qian and co-workers [31].) Our model considers the substrate to be mechanically rigid motivated by observations that it is in this limit that FAs are found to develop their maximum size [32]–[35].

An additional elastic contribution can arise from the change in cell membrane curvature as bonds form or dissociate at the proximal and distals ends of the FA. We let the bending modulus be denoted by 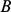 and the cell membrane's curvature be denoted by 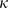. As depicted in Fig. 1 the cell membrane is straight over the FA domain, i.e., on 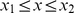. However, a curvature of 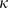 is possible for 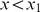 and 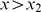. When FA growth takes place by binding of a new elastic element at 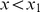 or 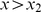, the cell membrane must be unbent over a length 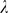 to change the curvature from 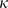 to zero. Unbinding allows the cell membrane to regain the curvature 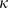. The free energy changes associated with stretching of elastic elements and bending/unbending of the cell membrane contribute to the total free energy change that drives FA dynamics, as will be shown below.

#### Complexity in focal adhesion dynamics

When FAs are subject to force by the contractile stress fibers, a positive feedback is created between the force and size of stress fibers, and FA size. The formation of an initial focal complex, to which they are anchored, allows the stress fibers to generate a force that in turn drives the further growth of stress fibers. Increased force can then be transmitted to the focal complex, causing it to grow into an FA by binding of ligand-integrin-plaque protein complexes. The process continues with growth of the FA and stress fibers. This feedback has been reviewed by Bershadsky and co-workers [36], and modelled by Besser and Safran [37] using reaction-diffusion equations and a force-controlled stress fiber growth model. In the present paper, however, we wish to focus on explaining FA growth in a constant force experiment independent of any feedback between the FA and stress fibers. Consequently we will not address this feedback. The emphasis here is on the specific thermodynamic mechanisms that enable the various regimes of FA dynamics.

FA dynamics will be modelled over a region 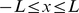, which is a line segment on the interfacial plane between the cell membrane and substrate as shown in Fig. 1. Since the FA's geometry evolves, the positions of its distal and proximal ends are time dependent, and are denoted by 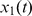 and 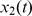, respectively. Its centroid is denoted by 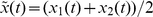 and its length is 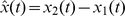. Its height remains fixed at 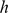, and it has width 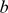 (not shown) in the interfacial plane along a direction perpendicular to the x-axis.

### Proximal and distal edge velocities lead to FA treadmilling, growth and resorption

**Figure 2:**
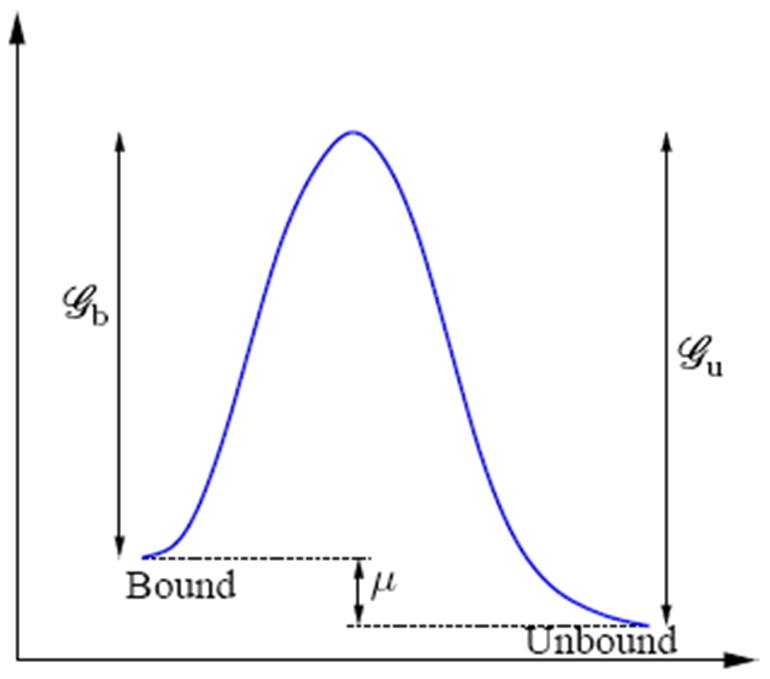
Schematic of the bound and unbound states represented by corresponding reaction coordinates, and the energy barriers to transitions between them.

**Figure 3:**
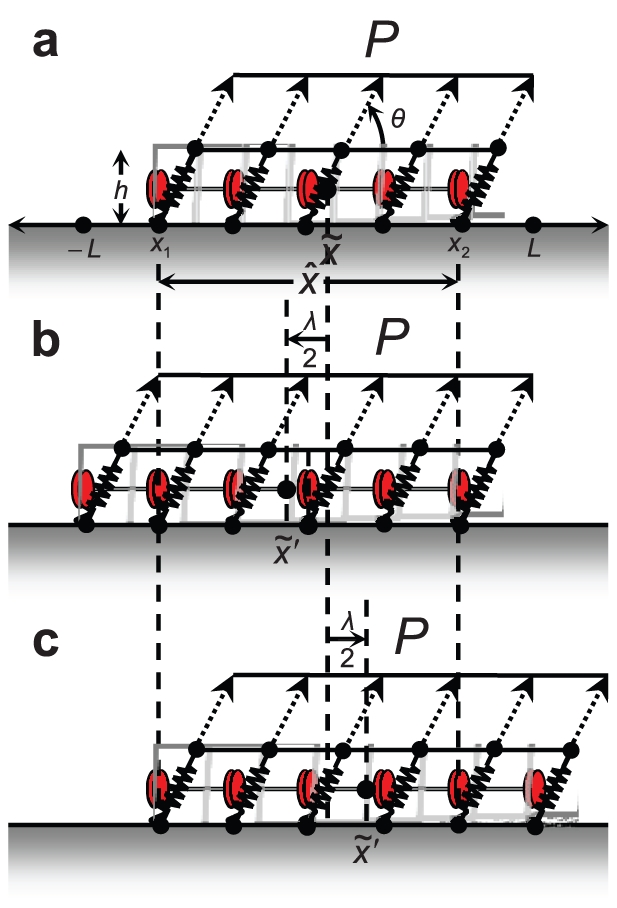
Schematic. (a) FA geometry and loading. (b) Addition of a complex at the distal end makes the center of the FA move opposite to the direction of the horizontal force component, increasing its potential. (c) Addition of a complex at the proximal end makes the center of the FA move in the direction of the horizontal force component, decreasing its potential.

FA growth at either end requires that one complex, which we estimate to have the average size, 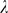, be added to attain a concentration 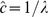 at the newly-formed end. At a binding rate 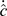, the time required to add a complex at the corresponding end is 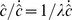. Over this time interval the corresponding edge of the FA advances by a distance 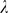. This gives the velocities of distal and proximal ends, respectively:
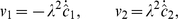
(1)respectively. For a force with positive 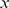-component as in Figs. 1, 2, 3, positive velocities, 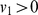, 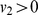, correspond to motion in the positive 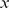-direction, and therefore to distal unbinding and proximal binding, respectively.

The Treadmilling Mode results when 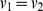 and 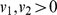. Proximal binding and distal unbinding causes the corresponding edges to move in the positive 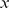-direction maintaining the FA size while the centroid translates in the force's direction. The Growth Mode is operative in all cases with 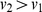. The Resorption Mode is operative in all cases with 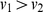. In the section titled “State diagrams of focal adhesion dynamics” we describe the development of these modes and their combinations. It is important to note that FA motion never happens with the same set of molecules making up the FA. Therefore, it does not move like a rigid body, but appears to do so as a combination of proximal and distal binding/unbinding.

### A rate process driven by non-equilibrium thermodynamics, and modulated by kinetics

The framework of Harmonic Transition State Theory, as proposed by Vineyard [24] underlies our theoretical treatment. Consider the binding and unbinding of complexes, as depicted in Fig. 2, applied to an elastic element located at any point along the FA. The net binding rate expressed in terms of number of complexes is
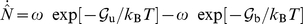
(2)where 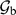 is the Gibbs free energy barrier between the bound state and the transition surface, 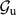 is the corresponding barrier between the unbound state and the transition surface, 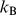 is the Boltzmann constant, 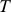 is the temperature, and 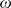 is an effective frequency associated with atomic vibration. We recognize that 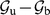 is the free energy difference between the binder-ligand pair in the bound and unbound states, and that this is also the chemical potential, 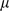, of the complexes. Accordingly we write,
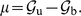
(3)


Suppose first that net binding occurs; i.e., 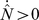 in Eq. (2). Using Eq. (3) it is easy to see that Eq. (2) can be written as
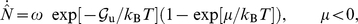
(4)with 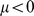 signifying that binding is thermodynamically favored. Next, consider unbinding; i.e., 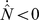. We also can write (2) as
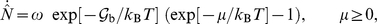
(5)signifying that unbinding is thermodynamically favored. Of course, all three forms (2), (4) and (5) give 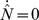 for 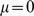; i.e., if the bound and unbound states are in thermodynamic equilibrium.

As proposed by Bell [28], the energy barrier to bond breakage, 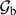 can be lowered by a suitably oriented force, a result which can be expressed as
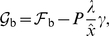
(6)where 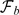 is the force-independent component of 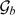. Here, we have estimated the force transmitted to the bond on a single complex to be 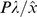, since the total force 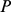 is spread over a length 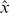. The parameter 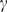 is the projection onto the force of the vector connecting the endpoints of the path along which the bond is deformed up to rupture. Following Bell's analysis we use 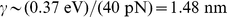. The unbinding rate therefore can be boosted by an exponential dependence on the force as shown immediately below. A recent paper [38] has reported a force-activated increase of the binding rate between integrins and ligands due to the hypothesized presence of catch bonds. Such an effect would be represented by an exponential dependence of the binding rate on 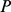 in Eq. (8)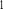.
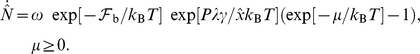
(7)


For the values listed in Table 1 this results in force activated unbinding kinetics at 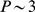 pN.

**Table 1:** Parameters used in the numerical examples.

Parameter (Symbol)	Value	Units	Remarks
Force-independent part of potential ( 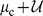 )	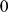	J	Models no growth for vanishing force.
Temperature ( 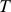 )	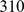	K	—
Actin stress fiber angle ( 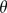 )	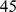	degrees	Effect of varying 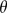 can be subsumed by varying 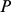 .
Young's modulus ( 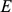 )	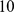	kPa	Estimate for soft, gel-like biological materials.
Bending modulus ( 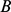 )	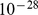	N.m 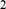	Estim. from 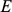 and cell memb. thickness 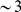 nm.
Cell memb. curvature ( 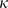 )	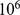	m 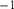	Estim. from cell height 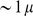 m.
Initial FA length ( 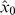 )	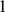	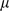 m	Typical focal complex length.
FA width ( 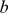 )	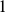	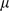 m	Estim. from images in [4] and [2].
Upper bound on complex size ( 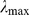 )	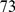	nm	Motivated by [29].
Lower bound on complex size ( 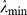 )	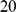	nm	Integrin packing density; see [30].
Conformational displacement ( 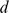 )	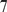	nm	Estimated from integrin domain size.
Kinetic coeff. ( 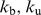 )	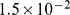	s 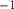	For sliding velocities 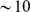 nm  s 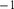 ; see [2].
Ref. displacement for unbinding ( 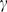 )	1.48	nm	From ref. [28].
Max. conc. ( 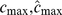 )	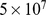	m 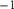	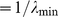 .

Eqs. (4) and (7) hold if there is no restriction on the local supplies of bound complexes and free binders, respectively. When the supplies are limited, the rates in Eqs. (4) and (7) must also be proportional to the concentrations of bound complexes and free binders, respectively. The rates therefore vary along the FA, and it proves convenient to express them as rates of change of concentration rather than of numbers. Since 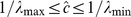, we note that the concentration of bound complexes is low for the parameters listed in Table 1, and a discrete treatment in terms of numbers of bound complexes rather than concentrations may be considered preferable on these grounds. However, we have found the ease of representing non-uniform fields via concentrations in Eq. (8) to be advantageous in this study. The proximal and distal edge velocities in (1) and the computed results also reflect this dependence on local concentrations. Finally we write kinetic coefficients for binding and unbinding, 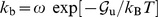 and 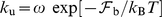, respectively. The rate equations are summarized below. (Rigorously, proportionality factors must be introduced in extending Eqs. (4) and (7), which express rates of change of numbers, to (8), which is a concentration rate. However, these factors can be absorbed into 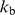 and 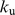.)
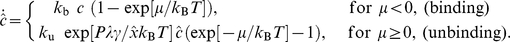
(8)


We note that a number of reactions are actually involved even in the simplified system considered here. These reactions include the binding/unbinding of ligands with integrins, of integrins with plaque proteins and of plaque proteins with actin. The single reaction rate of Eq. (8) can be viewed as representing the rate determining step of this cascade.

We now detail the specific thermodynamic driving forces that make up the chemical potential, 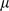.

#### Thermodynamic driving force from the work of complex addition at edges

Consider first the addition of a complex at the distal or proximal end to extend the FA domain. At time 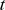 the distal and proximal ends of the FA are 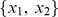. At time 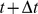 the FA is deemed to extend over the regions 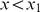 and 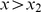 if the local concentration in these regions increases smoothly from 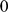 to 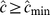 at time 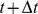. This concentration can be achieved by adding a single complex over the average length 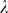. If the complex is added at the distal end the center of the FA shifts distally by 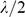. Conversely, addition of one complex over length 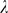 at the proximal end shifts the FA's center proximally by 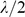. Since the newly-added complex bears some portion of the total force, 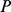, the result is a translation of the center of action of the force. Considering a force distributed uniformly over the FA for simplicity (but not necessity; computations including all the quantitative differences induced by non-uniform force distribution are also presented in this paper), the point of action of the total force translates with the FA centroid (see Fig. 3). A thermodynamic driving force arises due to the corresponding work done:
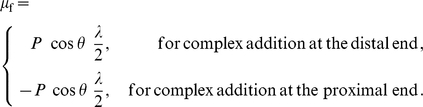
(9)The term, 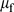, represents one contribution to the chemical potential, 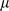. Its effect is antisymmetric with respect to complex addition at the distal and proximal ends. It is responsible for enabling the treadmilling regime of FA dynamics by favoring complex addition at the proximal end, but extracting an energetic cost for complex addition at the distal end.

#### Chemical driving forces

Let 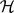 be the binding enthalpy. Using the concentration of free binders, 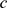, the mixing entropy is 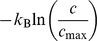. The chemical driving force that contributes to the potential is
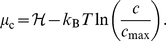
(10)Since bound complexes are immobile, they do not contribute to the mixing entropy or the resultant chemical potential. Also, consistent with the representation of the proteins in a cascade of reactions by a single free binder, we use a single mixing entropy term. Clearly, if 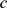 is equal between distal and proximal ends, 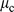 is also equal. In this case 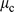 is symmetric between distal and proximal ends, and therefore does not influence FA treadmilling. This term causes symmetric binding if 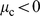 and conversely, unbinding for 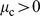.

#### Driving force due to elasticity

For uniform force distribution that has been assumed for simplicity of presentation (but not necessity; see the section titled “Sensitivity studies”, where a crack-like force distribution has been considered), the force acting on an elastic element of length 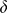 is 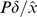. Since the elastic elements are modelled to have no shear stiffness as observed also by Bell [28], the ligand-integrin-plaque protein axis aligns with the force direction. The elastic free energy due to stretching of the element is
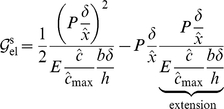


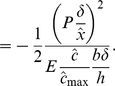
(11)As shown above, when the force is held constant the free energy is inversely proportional to the elastic modulus. The rigid substrate does not contribute an elastic free energy under force control since it has an effectively unbounded elastic modulus. In the above equation, the first term on the right hand-side of the first line is the elastic strain energy due to stretching of the elastic element, and the second is the change in potential of the force. This equation uses the fact that mechanical equilibrium is established at each instant for the current value of concentration 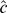. Using the number of complexes in the elastic element, 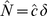, the elastic contribution to the chemical potential due to bond stretching is
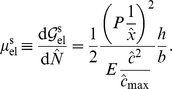
(12)


A further contribution comes from the elastic bending energy [39] as discussed in the section titled “The elasticity model”. Binding at the proximal or distal ends requires that the cell membrane be straightened out from its preferred curvature, 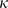, to zero curvature. The elastic free energy to straighten the cell membrane out over the length 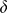 of an elastic element is
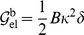
(13)


Using 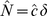 gives the contribution to the chemical potential due to bending of the cell membrane
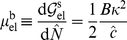
(14)


The total elastic contribution to the chemical potential is
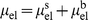
(15)


The length 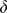 of the elastic element makes its appearance during the derivation of Eqs. (12) and (14). For this reason, the elastic stiffness used in Eq. (11) is proportional to 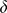. Note that stiffness is a structural parameter and includes the effect of size in contrast with the elastic modulus 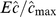, which is a material parameter and is independent of size. We point out that 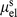 and 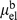 are independent of 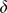, and therefore so are the resulting dynamics. The length, 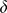, has no further role in the formulation. For equal concentrations, 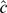, at the distal and proximal ends, these elastic contributions to the potential are also symmetric. Addition of a complex at either end results in the same value of 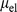.

For a given force distribution, a more sophisticated model for deformation of the FA, such as an elastic layer, would require the solution of a boundary value problem of linear elasticity with the additional complication of a concentration-dependent elastic modulus to determine stress distributions. While the quantitative results would change, the underlying physics would not be qualitatively different in our model. Alternately, in some studies [31], [40] arrays of FAs have been considered and fracture mechanics has been invoked to model the intervening gap regions between the cell membrane and substrate as cracks. With this approach a non-uniform stress distribution is obtained with stress concentrations at the distal and proximal edges. In the section titled “Sensitivity studies” we consider crack-like stress fields to demonstrate the model's robustness against variations in the stress distribution.

#### Driving force due to work done by force via conformational changes; protein mechanosensitivity

Conformational changes that are favorable to focal adhesion growth under tension have been hypothesized in vinculin [1], talin [7] and fibronectin [6] molecules as models of protein mechanosensitivity. Guided by these hypotheses we identify an additional possible driving force for complex formation under tensile force: Let 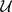 be the change in internal energy of the conformation-changing molecule. This is the contribution to the chemical potential due to the conformational change in the absence of force. Additionally, if an external tensile force is transmitted to this molecule, there is a lowering of the potential energy of the system consisting of the molecule and the force mechanism if the displacement vectors of the conformational change have components aligned with the force vector. Thereby, the free energy of the system is also lowered, and these changed conformations are thermodynamically favored under an external tension. The total contribution to the chemical potential from the conformational change then is written as
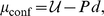
(16)where 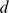 is the component of the conformational length change along 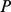. This term, like 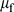, arises from work done by the force. While geometric details of conformational changes have been computed, e.g. for talin [7], they can be reduced to the form in Eq. (16). Like 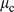 and 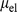 the term, 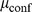, is symmetric with respect to distal and proximal ends if the force distribution is equal at the two ends. For this reason it also cannot be the universal mechanism that is responsible for FA treadmilling.

The combination of Eqs. (9–16) fully specifies the chemical potential:
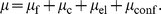
(17)


We emphasize that of the various contributions to the chemical potential only 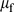 is antisymmetric between distal and proximal ends. The others, 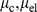 and 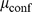 are symmetric between the ends. Therefore, while important to the total potential, 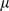, they do not cause symmetry breaking, which is necessary for the treadmilling mode.

The rate at which the concentration of bound complexes changes due to binding and unbinding can be computed, as described above, at any point on the FA. However, for the purpose of studying the growth and resorption of the FA structure, it is sufficient to focus on the distal and proximal ends only. This will be the aim in the remainder of this communication with special attention paid to symmetry breaking between the ends.

## Results

### Focal adhesion dynamics

**Figure 4:**
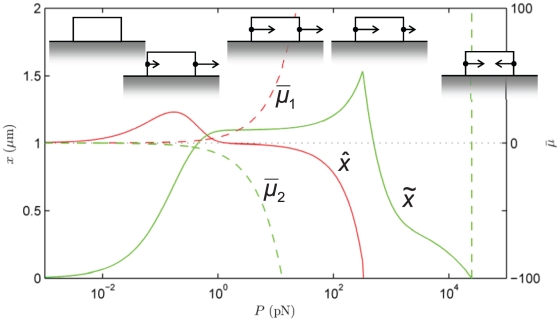
State diagram of the final position of the focal adhesion's centroid, 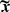, and length, 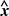 at 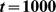 s as a function of force, 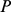. Also shown are the normalized chemical potentials, 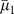 and 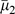 at the distal and proximal ends, respectively. The schematic diagrams indicate the dynamics corresponding to each regime. Note the various modes attained as 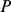 is varied.

**Figure 5 pone-0012043-g005:**
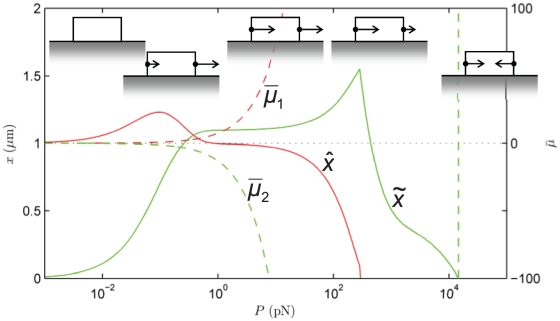
Effect of a crack-like force distribution, **Eq. (19)**. State diagram of the final position of the focal adhesion's centroid, 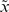, and length, 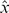 at 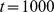 s as a function of *total* force, 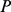. The other parameters are as in Fig. 4, with which this state diagram should be compared. Also shown are the normalized chemical potentials, 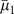 and 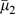 at the distal and proximal ends, respectively.

**Figure 6 pone-0012043-g006:**
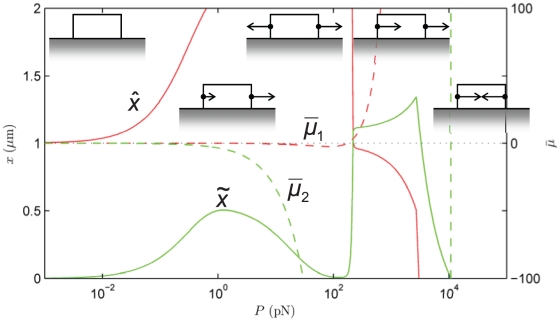
Sensitivity of the model to changing the complex size, from the upper bound 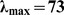 nm to the lower bound 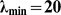 nm. The other parameters are as in Fig. 4, with which this state diagram should be compared.

**Figure 7 pone-0012043-g007:**
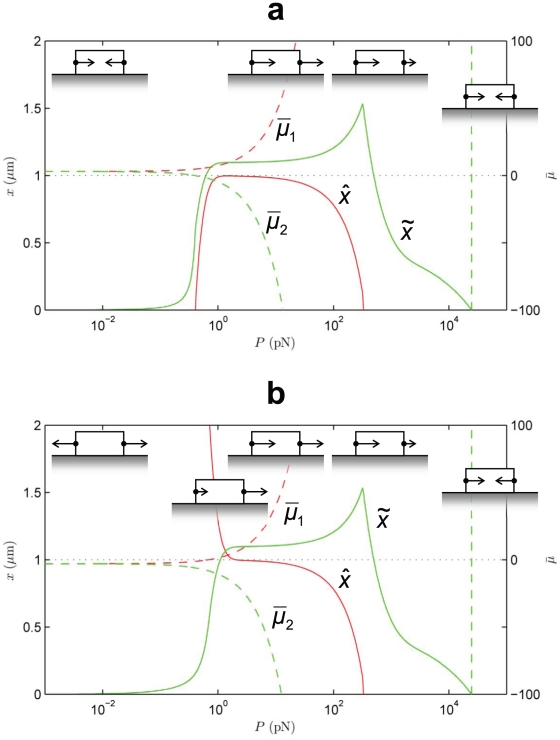
Sensitivity of the model to variation in the force-indpendent part of the potential, 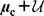. Here this combination has been varied between (a) 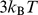 and (b) 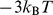. In comparison, 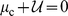 in Fig. 4. The other parameters are as in Fig. 4, with which this state diagram should be compared.

**Figure 8:**
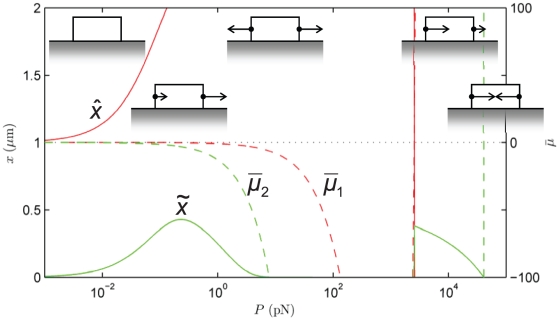
Sensitivity of the model to variation in the size of the conformational change, 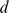. Here this parameter has been changed to 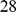 nm from 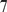 nm assumed in Fig. 4. The other parameters are as in Fig. 4, with which this state diagram should be compared.

Eq. (8) was solved numerically using the nonlinear ordinary differential equation integration routines in MATLAB. The parameters that appear in Table 1 have been fixed for the main study that follows (Fig. 4), but the model's sensitivity to some of them has been shown in Figs. 5, 6, 7, 8.

The following remarks can be made regarding our choice of parameters:

1. Eq. (10) identifies enthalpic and entropic contributions to the chemical part of the potential, 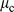, and Eq. 16 identifies a force-independent (and therefore, chemical) part of the conformational change induced potential, 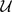. There are studies suggesting that the binding enthalpy of integrins to ligands lies in the range of 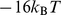 [41] to 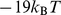 [42]; however, this does not account for the further step by which plaque proteins bind to integrin. It is therefore difficult to precisely evaluate the individual contributions to 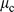 and 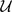. However, experiments suggest that FAs do not grow in the absence of force [2], [4], [10], a result that is attained in our model by setting 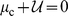, and that we have adopted for the studies that follow.
2. We have identified bounds on the complex size: 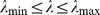. In generating numerical results with our model, we have first considered 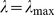, followed by 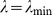 as part of a sensitivity study (Fig. 6).
3. Our choice of a numerical value for the conformational change, 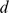 is guided by the sizes of integrin and plaque protein molecules in the FA. Our first set of calculations is with 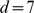 nm, motivated by the approximate size of the integrin domain. The resulting dynamics are not qualitatively different unless this value is increased to 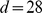 nm (Fig. 8)— a sensitivity study whose results we put into perspective by comparison with experiments.

#### State diagrams of focal adhesion dynamics

The observed dynamics at time 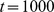 s are summarized in the state diagram of Fig. 4 by showing the FA length and displacement of its centroid on the left vertical axis *versus* the force, 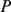 on the horizontal axis. This time instant has been chosen to correspond with the typical duration of a force-driven experiment [2], [4],[10]. Note that changes in length represent growth/resorption, while displacement of the centroid represents the effect of treadmilling. Also shown on the right vertical axis is the thermodynamic driving force represented by the normalized chemical potential, 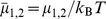 at the distal and proximal ends, respectively. The prevailing dynamic mode changes as the force is increased, and is indicated by schematic diagrams on which the direction and relative magnitudes of proximal and distal edge velocities are indicated by arrows. These modes are also identified in the text.

In the absence of force, i.e., when chemistry alone operates, 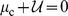 as argued above. The FA does not change in length (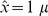
m) and its centroid remains stationary (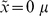
m). This is the Static Mode. As the force 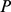, inclined to the right in Figs. 1 and 3, increases from zero, the different mechanical contributions to 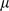 begin to exert their influences. The distal- and proximal-end potentials become respectively, positive and negative to favor unbinding and binding from a combination of the following mechanisms: (i) the antisymmetric work of complex addition, (ii) the symmetric elastic free energy, and (iii) the symmetric conformational change. However, the combination of mechanisms makes 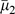 more strongly negative than 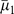 is made positive. As a result the proximal end binds more rapidly than the distal end unbinds. The resulting mode is Treadmilling with Growth. However, as the force increases and reaches 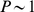 pN the distal edge unbinds as rapidly as the leading edge binds. The length, 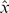 remains fixed at 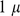
m while the centroid moves rightward following the force as demonstrated by 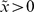. This is the pure Treadmilling Mode, which dominates until 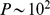 pN, with an unchanged amount of treadmilling. Over the range 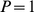
–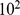 pN the Treadmilling Mode persists for arbitrarily large times since, according to the model, there are no changes in any of the contributions to 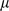 (see Movie S3).

Beyond this force the trailing edge unbinds more rapidly than the leading edge binds, and the FA length shrinks to 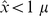
m. The distal unbinding is boosted in this regime due to the force-induced enhancement of unbinding kinetics as expressed in Eq. (8b). This is the Treadmilling with Resorption Mode. Between 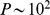 pN and 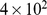 pN each increment in force causes an increment in treadmilling (see the increasing 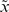 over this regime). However, force-induced enhancement of unbinding kinetics at the distal end causes 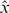 to decrease rapidly with force, and finally a transition sets in at 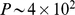 pN. The distal unbinding becomes so pronounced that 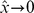, and the FA is fully resorbed. The amount of treadmilling also enters a steep decline with force. Finally, at 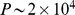 pN a thermodynamic transition also takes place. The thermodynamic driving force comes to be dominated by the quadratic elastic energy term, and causes unbinding at the proximal end in addition to the distal end. This is seen in the very sharp profile of 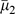 as it crosses into the positive half-plane. This is the Mechanical Resorption Mode in which thermodynamics and kinetics combine to cause very rapid unbinding at both ends; 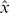 shrinks and the FA is fully resorbed very rapidly.

The appendix shows the corresponding state diagram if the stress on the FA is the controlled parameter instead of the force.

#### Inadequacy of linear response theory

Linear response theory of classical non-equilibrium thermodynamics as laid out by de Groot and Mazur [25] is easily recovered from Eq. (8) by expanding 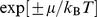 up to first-order in 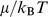. The linearized version of the rate law, Eq. (8) is
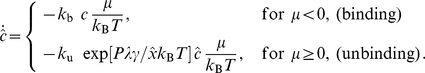
(18)


The form of Eq. (18) is a combination of Equation (18) in Chapter III, and Equation (18) in Chapter IV of de Groot and Mazur [25]. Their equations are in terms of the affinity, which is the product of a stoichiometric factor and 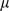. The stoichiometric factor reduces to unity in our case because of the generic reaction that we consider between one binder and one ligand. In the framework described by de Groot and Mazur, therefore, 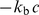 is the overall binding coefficient, and 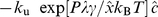 is the overall unbinding coefficient.

Note, however, that the first-order approximations in Eq. (18) hold only for 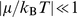. On examining Fig. 4, it is clear, therefore, that linear response theory is inadequate for describing FA dynamics since 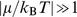 is required to access the relevant dynamic modes.

#### Sensitivity studies

Some authors [31], [40] have considered arrays of FAs and invoked fracture mechanics to model the intervening gap regions between the cell membrane and substrate as cracks. To demonstrate the model's robustness against variations in the stress distribution we address this case for the first of our sensitivity studies. On adapting the treatment of Lin and Freund [40] to our model the stress distribution along the FA is:
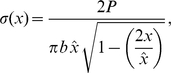
(19)


The resulting state diagram is Fig. 5, and is to be compared with Fig. 4. The differences are minor; the transitions between the different regimes occur at lower force, 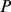, because the edge stress concentration causes the distal edge to unbind at lower force.

Fig. 6 demonstrates the model's sensitivity to variation of the complex size from the upper bound, 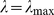 to the lower bound 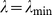. This weakens the antisymmetric work term due to complex addition, 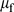 (Eq. (9)). When compared with Fig. 4 the main difference is that distal unbinding is weaker, as reflected by the delayed divergence of 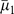. This allows the development of a Treadmilling with Growth Mode from 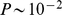 pN to 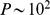 pN. This is followed by a Symmetric Growth Mode over a very small force regime, and a pure Treadmilling Mode over an even smaller force regime. From 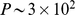 pN to 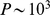 pN there is a Treadmilling with Resorption Mode. The decrease in 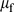, which is linear in 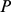 and competes against the quadratic elastic energy 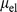, allows 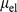 to make its influence felt at a lower value of 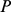. Therefore the thermodynamic transition to Mechanical Resorption occurs earlier for this particular choice of 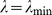.

Fig. 7 demonstrates the sensitivity to variation in the force-independent part of the potential, 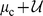 arising from the chemical part of the potential, and the internal energy of the conformational change, respectively. We consider this sum of terms to vary in the range of the thermal energy to model the spontaneous growth and resorption of the FA in the absence of force. We draw the reader's attention the changes in the low-force regime when compared with Fig. 4 : For 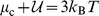 FA growth is not favored in the absence of force, causing resorption for forces 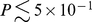 pN. Conversely, 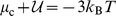 causes symmetric growth for forces 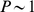 pN.

Fig 8 demonstrates the sensitivity of the model to the size of the conformational change, 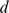. On increasing 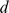 to 28 nm the chemical potentials at both ends become negative at low force, and binding is strongly favored at both ends. A Treadmilling with Growth Mode sets in at 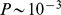 pN. However, due to the larger conformational change, a Symmetric Growth Mode develops at 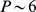 pN. This mode is strong enough that the centroid, 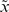, remains at its initial position, while the length, 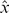, grows well beyond 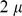
m. The Symmetric Growth Mode persists until 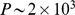 pN. The force-enhanced unbinding kinetics causes a transition to Treadmilling with Resorption at about the same force level as in Fig. 4, and is followed by the thermodynamic transition to the Mechanical Resorption Mode at marginally higher force than in Fig. 4.

In File S1 we have provided a list of movies of FA dynamics in the reaction-limited regime (Movies S1, S2, S3, S4, S5, S6, S7, S8), as well as movies of computations resulting from the extension of our formulation to the reaction-diffusion regime (Movies S9 and S10).

### Summary of results in relation to mechanisms

1. The central result of this study is that FA treadmilling is explained by the antisymmetric work done by the external force while complexes are being added to or removed from the FA. This mechanism provides the required symmetry-breaking in the thermodynamic driving force, and has the compelling feature that the most elementary concept of Work is sufficient to explain treadmilling. There is no need to invoke special constitutive hypotheses for this purpose. We recognize, however, that it does not rule out such constitutive models (see “Relation to other models in the literature”). On the other hand the symmetry of the chemical, elastic and conformational change-driven components of the chemical potential makes them unlikely contenders for the role of the mechanism that enables treadmilling.
2. Once the symmetry of the thermodynamic driving force has been broken, the kinetics lead to further asymmetry due to the force dependence of the unbinding rate. The interplay between the kinetic and thermodynamic effects determines the growth, treadmilling and resorption modes in Figs. 4, 5, 6, 7, 8.
3. The elastic part of the chemical potential has one contribution, 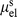, which is quadratic in force and contributes to thermodynamically-driven resorption of the FA. We have found that the elastic contribution due to cell membrane bending, 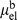, has no noticeable effect on the dynamics, and is dominated by the bond stretching contribution, 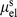.
4. Figs. 4, 5, 6, 7, 8 emphasize the dynamic behavior of FAs under the influence of force. However, we note that the model does not preclude the existence of a static FA under force. This only requires a non-uniform force distribution over the FA by which the chemical potential vanishes at both ends: 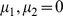.
5. The dominant direction of force exerted by the actin stress fibers is along the axis of the FA. However, there are reports of individual filaments having non-axial orientations in the plane of the FA [43]. The conclusion reached with our model is that the force transmitted by these individual filaments does result in growth along nonaxial (e.g., widthwise) directions in the FA plane. However, since the dominant direction of actin stress fibers is axial, the FA grows substantially more in the axial direction and attains an elongated shape. We have modelled the idealized case of constant width, 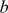.
6. We have tested several different forms of the rate law in place of Eq. (8). Some of these forms have reached saturation at finite rates as 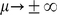, others have had no exponential dependence on force 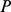, and yet others have been linear in 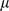. All these forms were found to be qualitatively equivalent in the sense that the state diagram Fig. 4 showed the same dynamic modes, and the sensitivity studies in Figs. 5–8 demonstrated the same changes in these modes. In this sense the results and our conclusions reported in this section are independent of the rate law. The quantitative results were, of course, different in that the transitions between different modes occurred at different force levels for these different rate laws. The kinetic coefficients 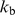 and 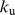 were also different for these different rate laws. The results comparing different rate laws have not been shown for the sake of brevity.

## Discussion

### Comparison with experiments

1. The distinct modes of focal adhesion growth, treadmilling, resorption and combinations of these modes are obtained as the force is varied. The dynamics seen in the supporting movie of Nicolas and co-workers [8] and in Aroush and co-workers [10] is well-represented by the model.
2. The state diagrams in Fig. 6 with 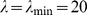 nm, and Fig. 8 with 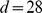 nm include the Symmetric Growth Mode, in which distal and proximal ends grow outward at the same velocity. This mode, however, has not been observed in experiments [2], [4], [10], suggesting that these parametric values are not representative of the underlying physics.
3. Fig. 6, shows that the FA remains robust at 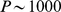 pN. For a FA of area 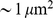 this leads to a stress 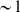 kPa, a prediction which corresponds well with the stress of 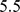 kPa measured by Balaban and co-workers [4]. Since this state diagram was generated using the smaller size 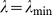 for the ligand-integrin-plaque protein, it suggests that this value is more representative of the physics than 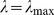.
4. As the second more specific prediction we note the following: For a cell radius of 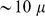
m our finding that FAs are resorbed at 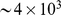
–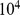 pN (Figs. 4 and 5) suggests that 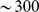
–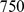 FAs are needed to attain a cell adhesion strength 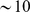 kPa measured by Garcia and co-workers [44].
5. For the third prediction we note: If a single ligand-integrin-plaque protein complex has size 20–73 nm and is attached to a single actin filament, between 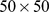 and 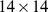 filaments transmit force to an FA of size 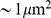. This requires that at a force that causes FA resorption, 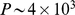
–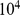 pN, each actin filament can bear 1.6–50 pN force without itself breaking. These levels are far below the 200–600 pN rupture force of actin filaments measured using glass needle micromanipulation *in vitro* by Tsuda et al. [45], and even typical loads of 120–200 pN in the muscle sarcomere during isometric contraction by Oosawa [46]. Separately, rupture forces for standalone 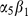 integrin-fibronectin complexes were 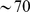 pN as measured via AFM [47]. Therefore the force level required in the actin filaments by our model is within experimental bounds.
6. Recent experiments [48] have shown that when fibroblasts are plated on compliant substrates that are dynamically stretched, the FAs in the fibroblasts orient with the direction of stretching. Our model is able to explain this observation when we carry out a two-dimensional extension of the antisymmetric work term, 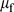. We have 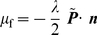, where 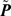 is the projection of the force vector 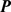 on the interfacial plane, and 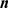 is the unit vector in the interfacial plane along the direction of FA extension/retraction due to complex binding/unbinding. Clearly, 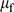 is most negative when 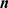 is aligned with 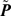, as happens for proximal binding and distal unbinding in the direction of 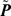. Conversely, 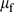 is most positive when 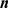 is aligned opposite to 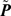 in the interfacial plane, as happens for proximal unbinding and distal binding in the direction opposite to 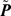. Therefore, proximal binding and distal unbinding are favored in the direction of 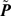, and the FA aligns with the force projection 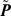.
7. In experiments carried out by Cluzel et al. [49] the integrins from FA structures remained intact even after the transmission of force was disrupted by destruction of the actin cytoskeleton. However, proteins such as paxillin and vinculin were not recruited in the absence of force. Since the different proteins are not explicitly modelled, this detail is not represented in our results. Instead, our model's results represent the failure to grow the FA structure in the absence of force. This is in correspondance with the experimental observations of Cluzel et al.
8. We have considered the effects of variation of a few parameters on the state diagrams in Figs. 4–8. These include the force distribution, the complex size 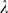, the force-independent part of the potential 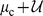, and the conformational change 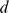. Through these studies we have identified broad regimes and trends described in these state diagrams, argued against some parametric values, made specific predictions, and made connections to additional experiments. Molecular structural studies and conformational changes predicted by molecular modelling will bring greater certainty to estimates for 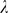 and 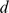. With better estimates for parameters, force-controlled loading of individual FAs, e.g. by actuating micropillars, could provide further benchmarks against which specific predictions of our model can be tested, and the model can be verified or falsified.

### Relation to other models in the literature

In general, the papers that we review in this section have addressed the dynamics of FA response to force. Growth, treadmilling and resorption regimes have been reproduced by some of these models. Our contribution, coming after these papers, is focused on alternate, perhaps simpler, explanations of FA dynamics.

Shemesh and co-workers [16] and Besser and Safran [17] (the latter paper encompasses and elaborates upon earlier work by Nicolas and co-workers [8] and Nicolas and Safran [15]) have attempted to explain FA dynamics in thermodynamic or kinetic terms, although the details of each treatment are different from ours. The most significant manifestation of these differences is the conclusion reached in these two papers that as the external force on the FA increases beyond some level, the FA continues to grow, and does so symmetrically. In contrast our study concludes that the force-induced enhancement of unbinding kinetics, and the quadratic force-dependence of the elastic free energy ultimately cause resorption (Figs. 4–8). We note that the more recent work of Nicolas and co-workers [19] reaches the same conclusion as we do with regard to the ultimate resorption of the FA at high force levels.

Shemesh and co-workers require a very specific geometry of the FA: free of ligand-integrin bonds at the proximal end, and bonded at the distal end. Without such a geometry the gradient of FA stress that they find to drive transport disappears and their model fails. Also see the comments by Besser and Safran [17] in this regard.

Besser and Safran [17] and Nicolas and co-workers [19] consider the detailed geometry pf the integrin-ligand bonded region: extending slightly beyond the plaque protein layer distally and proximally. (Also see the appendix of this communication where this geometry is used to explain symmetry-breaking by the work done during addition/removal of a bound complex under stress control.) The authors hypothesize that the proximal compressive stress in the integrin-ligand bonded layer induces a molecular conformational change that favors FA growth via binding, while the distal tensile stress inhibits binding, and therefore downregulates growth. However, all the molecular conformation changes that have been hypothesized to favor FA growth [1], [6], and investigated [7] involve uncoiling by local tension, not compression. Compression in one direction can induce tension in any other direction according to the theory of elasticity. However, a negative Poisson ratio and a constraint against free deformation in the second direction are both required. These make up rather specific constitutive and microstructural assumptions, which have not been investigated to our knowledge. Nicolas and co-workers [19] consider the possibility that a force-induced gradient in conformation also may favor binding. Apart from the possible roles of compression and gradient in conformation, the treatment of the conformational change by Besser and Safran [17] and by Nicolas and co-workers [19] is essentially the same as ours.

We note that Shemesh and co-workers [16] applied the Gibbs-Duhem relation to obtain a result for potential due to elasticity that is equivalent to our elastic free energy results in Eqs. (11) and (13). However, they did not consider the change in stiffness with concentration, for which reason their elastic potential is the same as our elastic free energy. Also, in contrast with their model and the model of Besser and Safran [17] or of Nicolas and co-workers [19], our model does not include in-plane shear stress, because we have neglected shear stiffness—using a physical argument on orientation of integrin-ligand bonds that was also invoked by Bell [28]. While Nicolas and co-workers [19] have considered FA dynamics dependent on substrate stiffness, our study considers a mechanically rigid (infinitely stiff) substrate that does not store elastic energy under force-control, and therefore does not affect the thermodynamics. It is in this limiting case that FAs develop to their maximum size [32]–[35].

Deshpande and co-workers [18] have built in a finite strength via an elastic potential for the integrin-ligand bonds. In the present study, however, a finite strength emerges from competition between different thermodynamic driving forces for binding and unbinding, modulated by force-enhanced kinetics. Deshpande and co-workers' constitutive model assumes integrin sliding once the maximum stress is reached. They discuss this mechanism qualitatively to rationalize their constitutive model. In constrast we have presented an integrated quantitative treatment that accounts for FA growth, treadmilling and resorption. Deshpande and co-workers consider low- and high-affinity states of integrin molecules that correspond to the unbound and bound states in our work. They advance two distinct and independent hypotheses for the sliding mode (equivalent to our treadmilling mode) of FA dynamics: (i) Integrin molecules switch from high affinity states of deformed, energetically-unfavorable conformations into low affinity and low energy states. Other integrins maintain strain continuity of the FA by switching from low to high affinity, but lower energy, states. (ii) The propagation of a front of switching from high to low affinity states allows FA sliding—a mechanism that they liken to dislocation glide in crystals. These are highly specific mechanisms that await validation.

Aroush and co-workers [10] have studied the accumulation and depletion of proteins over individual FAs. They observe growth, treadmilling and resorption, and combinations of these modes, all of which are reproduced by our model. Direct comparisons are not possible because their experimental study did not employ force control, which is the case that we have considered. Instead, the FAs in their study were subject to force from actomyosin contractility. Cytoskeletal dynamics therefore affected the magnitude of force developed. However, their study did bear out the predominantly one-dimensional nature of FA dynamics, which is the case we considered. Aroush and co-workers also developed a model for the stress distribution along the FA-ECM interface based on shear-lag effects. They make the constitutive hypothesis that the unsymmetric stress profile that emerges as a main result of shear lag could be responsible for causing unbinding of proteins at the distal edge. They do not consider fundamental physical mechanisms that could favor binding or unbinding as we have done.

Importantly, the present communication finds no need for special constitutive assumptions on the response characteristics of the material in a FA to explain the treadmilling regime. Instead, treadmilling is explained by a more fundamental principle: It is driven by the asymmetry between the work done when complexes are added at the distal and proximal ends, respectively. If the findings reported here stand up to experimental validation they could be the basis for techniques by which to control FA formation by interfering with force generation of stress fibers in actin-targeted therapies. Furthermore, this model can be combined with models of cytoskeleton dynamics and contribute to the understanding of cell adhesion and motility.

### Appendix: The stress-controlled case

Under stress control the appropriate thermodynamic potential differs from the Gibbs free energy, 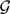. Instead of 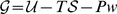, with internal energy 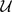, entropy 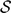 and displacement 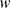, we have
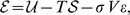
(20)where 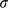 is the stress, 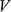 the volume and 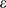 is the strain. In analogy with Fig. 2 we write 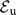 as the barrier between the unbound state and the transition state, and 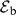 as the barrier between the bound state and the transition state, respectively. Proceeding as in Eqs. (2–8) with 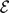 taking the place of 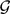 we have
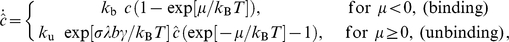
(21)where the chemical potential is redefined as 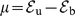 and 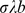 is the force on a bound complex. The other quantities have the same definitions as in Eqs. (2–8).

It remains now to specify the contributions to 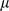. From Eq. (20) it follows that the addition of a complex of length 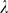 at the distal or proximal edge changes 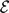 as follows: 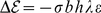. Since 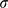 is the applied stress, any non-uniformity in this quantity could induce symmetry-breaking between the proximal and distal edges. Even if 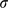 is equal at the two edges the sign of this contribution depends on 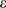. Recall from the first paragraphs of the Introduction and the section titled “A reaction-limited process” that some plaque proteins bind with integrins and others bind to F-actin. Since a substrate must be available for newly-attached actin to generate force, it suggests that at least some assembly of plaque proteins must happen before actin binds to them. If this newly forming complex, with plaque proteins but no bond to actin yet, is at the proximal edge it is compressed by the actin-loaded FA behind it; alternately, if it forms at the distal edge it is stretched by the actin-loaded FA ahead of it [15]. Once the actin bond forms this complex gets stretched by the stress 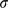; however, proximal stretching, 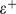, is from a compressed reference state, while the distal stretching, 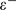, is from an already-stretched reference state. On this basis we have 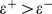 if 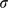 is uniform. The associated change in 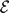 is the chemical potential from the work of complex addition at edges:
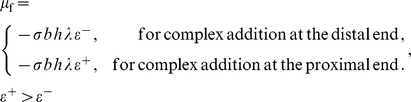
(22)


This is the symmetry-breaking mechanism under stress-control. The details of how it operates do differ from symmetry-breaking under force-control (Eq. (9)), and the mechanism summarized in Eq. (22) does require some shear stress to develop; see Ref. [15]. However, this is to be expected. Since the boundary conditions on the system have changed, its response also has changed and effects that were neglected for force-control must now be included. Importantly, the feature of symmetry-breakage remains.

Since the cases of force- or stress-control introduce no differences in the chemical enthalpy or the entropy, the contribution from chemical driving forces is the same as in Eq. (10):
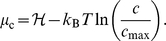
(23)


Following the development of the section titled “Driving force due to elasticity”, but accounting for stress-control instead of force-control, we have
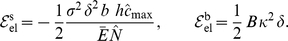
(24)


Using 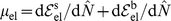 and 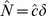 gives
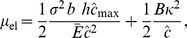
(25)where 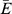 is an effective modulus for the combined normal-shear mode of deformation.

The driving force due to work done by conformational change of a protein is obtained by following the arguments to Eq. (16) and replacing the force 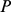 with 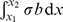:
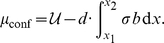
(26)


**Figure 9:**
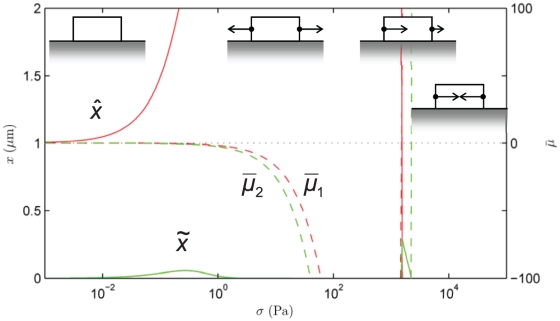
State diagram of FA dynamics for stress-control (see Appendix). The strains used were 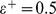, 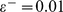, with other parameters are as in Fig. 4, with which this state diagram could be compared. The main effect of stress-control on FA dynamics is that the symmetric growth mode is strongly favored over most low stress values, as seen by the growth of 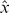. However, the Treadmilling Mode does hold over a narrow range for 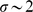 kPa, which compares well with the stress of 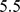 kPa reported by Balaban and co-workers [4]. The Treadmilling Mode gets suppressed for less polarized strains 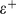 relative to 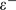.

Then the total chemical potential from all four mechanisms is 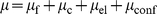 using Eqs. (22–26). Note that for a uniform stress, 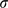, the only symmetry-breaking mechanism is the work done due to complex addition at the edges, and therefore this is the mechanism that largely determines the treadmilling regime. A state diagram of FA dynamics under stress-control appears as Fig. 9. While the Symmetric Growth Mode dominates the dynamics, there is a small regime of the Treadmilling Mode at 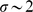 kPa, which correspnds well with the stress 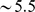 kPa measured by Balaban and co-workers [4]. Thus, the broad qualitative conclusions do not differ in the stress-controlled case, although the details of the regimes of the various dynamic modes do differ in quantitative terms. This is perhaps not surprising: the system differs in its response for different boundary conditions.

## Supporting Information

File S1 (0.02 MB PDF)

Movie S1 *P* = 0 pN and small conformational change; no growth.
(0.37 MB AVI)

Movie S2 *P* = 1×10^−1^ pN and small conformational change; treadmilling with growth.
(0.47 MB AVI)

Movie S3 *P* = 1×10^2^ pN and small conformational change; pure treadmilling.
(0.41 MB AVI)

Movie S4 *P* = 10^4^ pN and small conformational change; treadmilling with resorption.
(0.20 MB AVI)

Movie S5 *P* = 0 pN and large conformational change; no growth.
(0.37 MB AVI)

Movie S6 *P* = 1×10^−1^ pN and large conformational change; treadmilling dominated by growth at both ends.
(0.47 MB AVI)

Movie S7 *P* = 1×10^2^ pN and large conformational change; symmetric growth.
(0.42 MB AVI)

Movie S8 *P* = 10^4^ pN and large conformational change; treadmilling with resorption.
(0.20 MB AVI)

Movie S9 Reaction-diffusion formulation with *P* = 10^2^ pN and small conformational change; pure treadmilling is discernible.
(0.89 MB AVI)

Movie S10 Reaction-diffusion formulation with *P* = 10^2^ pN and large conformational change; treadmilling is dominated by growth at both ends.
(0.92 MB AVI)
