# Supplementary material for: The Non-Equilibrium Thermodynamics and Kinetics of Focal Adhesion Dynamics
Source: PLoS One. 2010 Aug 18;5(8):e12043. doi: 10.1371/journal.pone.0012043 (PMC2923603; doi:10.1371/journal.pone.0012043)
Supplement: File S1 — (0.02 MB PDF) [file pone.0012043.s001.pdf]

Supporting information

Reaction-limited formulation: The FA is in green. Only edge motion is shown for purposes of visualization; the changing concentration profile over the FA is not shown.

Movie S1: $P = 0$ pN and small conformational change; no growth.

Movie S2: $P = 1 \times 10^{-1}$ pN and small conformational change; treadmilling with growth.

Movie S3: $P = 1 \times 10^{2}$ pN and small conformational change; pure treadmilling.

Movie S4: $P = 10^{4}$ pN and small conformational change; treadmilling with resorption.

Movie S5: $P = 0$ pN and large conformational change; no growth.

Movie S6: $P = 1 \times 10^{-1}$ pN and large conformational change; treadmilling dominated by growth at both ends.

Movie S7: $P = 1 \times 10^{2}$ pN and large conformational change; symmetric growth.

Movie S8: $P = 10^{4}$ pN and large conformational change; treadmilling with resorption.

Reaction-diffusion formulation: The changing concentration profile over the FA is shown in green.

Movie S9: Reaction-diffusion formulation with $P = 10^{2}$ pN and small conformational change; pure treadmilling is discernible.

Movie S10: Reaction-diffusion formulation with $P = 10^{2}$ pN and large conformational change; treadmilling is dominated by growth at both ends.
